# Supplementary material for: Infraspinatus Fascial Dysfunction as a Cause of Painful Anterior Shoulder Snapping: Its Visualization via Dynamic Ultrasound and Its Resolution via Diagnostic Ultrasound-Guided Injection
Source: Diagnostics (Basel). 2023 Aug 4;13(15):2601. doi: 10.3390/diagnostics13152601 (PMC10417446; doi:10.3390/diagnostics13152601)
Supplement: Supplementary file 1 [file diagnostics-13-02601-s001.zip › diagnostics-2457639-supplementary.pdf]

Supplementary materials

for Infraspinatus Fascial Dysfunction as a Cause of Painful Anterior Shoulder Snapping: Its Visualization by Dynamic Ultrasound and Resolution by Diagnostic Ultrasound-Guided Injection

King Hei Stanley Lam [1,2,3,4,5,*], Daniel Chiung Jui Su [6], Yung–Tsan Wu [7,8,9], Mario Fajardo Pérez [10,11,12], Kenneth Dean Reeves [13], Philip Peng [14] and Bradley Fullerton [15,16]

Video S1. This video showed the clinical test by the treating physician. Resisted shoulder flexion with the elbow straight and the shoulder flexed at 90° with the internally rotated humerus (thumb down, similar to the empty can sign), both straight and abducted shoulder, had normal power and no pain. However, the power of resisted shoulder flexion with the elbow straight and the shoulder flexed to 90° with the humerus externally rotated (palm up) was diminished and associated with pain. Available online: https://www.dropbox.com/s/ll0m9spscvl0s8y/shoulder%20flexion%20against%20resistance_Weak.mp4?dl=0 (accessed on 1 January 2023).

Video S2. Dynamic ultrasonography of this patient in this video showed a thickened, fibrotic bursa (as shown by the yellow arrow in the video) snapped between the subscapularis and the short head of the biceps during external and internal rotation of the humerus, with the humerus in the anatomical position but elbow flexed and palm up to rotate the humerus internally and externally (the position of the shoulder under scanning was the same as that shown in Figure 1). The sonoanatomy of the involved structures are labeled in the still images embedded in the video. In the last part of the dynamic ultrasonography, there

was a cortical break over the right humeral head deep to the subscapularis tendon (as pointed out by the orange arrow). It looked like the subscapularis tendon over the cortical break did not have disruption in the compact fibrillar pattern and the cortical break over the humeral head is likely inside the joint capsule (Figure 1). Available online: https://www.dropbox.com/s/udsvs3bk5yh88eb/Anterolateral%20painful%20snapping%20combined.mp4?dl=0 (accessed on 1 January 2023).

Video S3. This video showed the dynamic ultrasound scanning with the humerus externally rotated (palm up), elbow straight and the shoulder actively flexed and abducted at about 90 to 100 degrees, the fibrotic and thickened bursa was noted to be snapping between the subscapularis tendon and the coracoacromial ligament. The colored still images with labelling embedded in the video described the sonoanatomy of the involved structures. Available online: https://www.dropbox.com/s/mqg7o8qf5ae9yfm/Snapping%20of%20subscapularis%20vs%20CAL.mp4?dl=0 (accessed on 1 January 2023).

Video S4. Ultrasound-guided injection of the painful anterior shoulder has been performed in twice. In each session, the needle touched the fibrotic bursa did not reproduce his usual pain, and the injection of the bursa did not relieve his pain or reduce the snapping. When the needle touched the cortical break and the bone, it reproduced patient's concordant pain. Immediately post-injection, the NRS dropped to 3/10; however, right shoulder snapping and flexion power were unchanged, and the pain recurred within one day. Available online: https://www.dropbox.com/s/cypx56rifefj5ka/Video%204%20new%20cortical%20break%20injection%20.mp4?dl=0 (accessed on 1 January 2023).

Video S5. This video showed detailed musculoskeletal examination of the scapular movement with the right scapulothoracic movements compared not as smooth as the left counterpart [4,5]. Still images of the suspected lesion sites were embedded in the video with labelling. Disruptions in the right infraspinatus fascia (IF) and related muscles were suspected, including the lateral edge of the right inferior trapezius, rhomboid minor and major [6], teres major, latissimus dorsi [7,8] (LD), and posterior deltoid attachments to the IF [9,10]. Available online: https://www.dropbox.com/s/eq7w9gksly83z09/Scapulothroacic%20movesment.mp4?dl=0 (accessed on 1 January 2023).

Video S6. Sonoanatomy of the lateral border of the inferior trapezius, its related muscles and infraspinatus fascia. The essential technique of scanning these structures is to follow the direction and contour of the ribs. The detailed sonoanatomy of the structures related to the lateral boarder of the trapezius were shown clearly in the paused images of the video and also in Figure 2. It is our observations that the lateral edge of the inferior trapezius usually leaves medial edge of the scapula at 6th rib and at 7th rib, the lateral edge of the inferior trapezius usually at the position midway between the spine and the medial edge of the scapula. Available online: https://www.dropbox.com/s/o086j8b3pimflpt/lateral%20border%20of%20trapezius.mp4?dl=0 (accessed on 1 January 2023).

Video S7. Sonoanatomy of the lateral border of the scapula and its related muscles and infraspinatus fascia. The detailed sonoanatomy of the structures related to the lateral edge of the scapula were shown clearly in the paused images of the video and also in Figure 3. It is worthwhile to note that the position of the structures related to the lateral edge of the scapula will be changed when the shoulder has different position because the muscles attached to the scapula will be stretched with the shoulder in abduction and flexion. Available online: https://www.dropbox.com/s/htrdpjjd8hyt9rl/Lateral%20edge%20of%20the%20scupla.mp4?dl=0 (accessed on 1 January 2023).

Video S8. Sonoanatomy of the medial edge of the scapula and its related muscles and infraspinatus fascia. The detailed sonoanatomy of the structures related to the medial boarder of the scapula were shown in the paused images of the video and also in Figure 4. It is also advisable to scan the medial edge of the scapula by the transducer in line with the ribs. The position of the structures related to the medial border of the scapula will also be changed when the shoulder is in different position. Available online: https://www.dropbox.com/s/7wxn4391aiivgt5/Medial%20Borader%20of%20Scapula.mp4?dl=0 (accessed on 1 January 2023).

Video S9. Sonoanatomy of scanning the infraspinatus fascia in the sagittal plane from rhomboid minor laterally and back to rhomboid major. The detailed sonoanatomy of the structures related were shown in the paused images of the video and the Figure 5. It is also sensible to note that the exact location of the structures related to the lateral edge of the scapula will be changed when the shoulder has different position because the muscles attached to the scapula will be stretched with the shoulder in abduction and flexion. Available online: https://www.dropbox.com/s/kr8sddnz7zm7wsc/Infraspinatus%20fascia%2C%20sagittal%20view.mp4?dl=0 (accessed on 1 January 2023).

Video S10. In this video, sonopalpation is performed along the infraspinatus fascia (IF) at the lateral edge of the scapula, from the inferior angle to the humerus and back to the inferior angle. The muscles of interest include infraspinatus, teres minor and posterior deltoid; also visible are teres major and the superior edge of latissimus dorsi. The sonopalpation was then repeated on the sound side for comparison. In this video, the painful shoulder exhibited laxity of the IF with excessive displacement of the teres minor and the adjacent infraspinatus fibers, the medial edge of the posterior deltoid and the teres major near the inferior angle. These are signified by the red arrow; the normal left counterparts are indicated by the yellow arrow. Available online: https://www.dropbox.com/s/xf5xyopgmjaczuc/Lateral%20edge%20of%20scapula%20sonopalpation_name%20ad%20arrow.mp4?dl=0 (accessed on 1 January 2023).

Video S11. Example of ultrasound-guided digital palpation of the edge of the scapula for tenderness and loss of resilience of the IF. The targets of the palpation were put in the centre of the transducer and the palpating finger of the physician palpate just next to the centre of the transducer, especially the edge of the scapula for any excessive displacement of the soft tissue of the IF as compared to the sound side. The excessive displacement might be visualized with the ultrasound. However, when the finger has been inserted between the transducer and the skin, it will block the view of the structures under palpation. The ultrasound-guided digital palpation has some benefit over the transducer sonopalpation as the finger of the physician is usually more precise and sensitive when comparing to the ultrasound transducer itself. This may be useful at locations where bone is deeper under the skin, such as at the lateral edge of the trapezius before it crosses the medial border of the scapula. However, this also adds another operator dependent factor to the limitation or difficulty of this technique. Available online: https://www.dropbox.com/s/glmjgc9bi94f41x/Digital%20palpation%20of%20the%20Edges%20of%20the%20scapula%20and%20trapezius.mp4?dl=0 (accessed on 1 January 2023).

Video S12. Video showed the ultrasound-guided diagnostic and therapeutic injection of 15% hypertonic glucose mixed with 0.1% lidocaine, targeted at the teres major and latissimus dorsi attachments on the inferior edge of the infraspinatus fascia. Available online: https://www.dropbox.com/s/71zcy9zedgf28sc/IF%20injection%20from%20Teres%20Major%20and%20Lat%20Dorsi.mp4?dl=0 (accessed on 1 January 2023).

Video S13. Video illustrated the ultrasound-guided diagnostic and therapeutic injection of the suspected lesions over lateral edge of the inferior trapezius attachments to medial side of the infraspinatus fascia. Available online: https://www.dropbox.com/s/xwm4g6pc3ddn6ao/IF%20injection%20near%20scapular%20spine_labeled.mp4?dl=0 (accessed on 1 January 2023).

Video S14. Video demonstrated the ultrasound-guided diagnostic and therapeutic injection of the hypertonic glucose with local anesthetics to another lesion at the lateral edge of the inferior trapezius attachments to medial side of the infraspiatus fascia. Available online: https://www.dropbox.com/s/uqr3gypt8fa1nqf/IF%20injection%20near%20inferior%20angle%20of%20scapula_labeled.mp4?dl=0 (accessed on1 January 2023).

Video S15. Video represented the ultrasound-guided diagnostic and therapeutic injection of 15% hypertonic glucose mixed with 0.1% lidocaine, aiming at the suspected defects at the superolateral attachment of the infraspinatus above the teres minor and major. Available online: https://www.dropbox.com/s/pm3e6exr73qj5n8/Teres%20Major%2C%20minor%20IF%20injection_labeled.mp4?dl=0 (accessed on1 January 2023).

Video S16. Video shows immediately post injection, the scapulothoracic movement of the shoulder improved significantly and was more symmetrical to the left scapulothoracic movement. Available online: https://www.dropbox.com/s/uhqd1t7odlvxtf9/Immediate%20post%20injection%20scapulothoracic%20movement_labeled.mp4?dl=0 (accessed on1 January 2023).

Video S17. Immediately post IF injection, the snapping between the thickened and fibrotic bursa between the subscapularis and short head of biceps completely resolved. The pain has also been relieved. The cortical break on the humeral head was still the same. Available online: https://www.dropbox.com/s/fulodr2b62bzf2c/Np%20Snapping%20of%20shoulder%20post%20injection.mov?dl=0 (accessed on1 January 2023).

Video S18. Video showed the power of resisted shoulder flexion with the elbow straight and the shoulder flexed to 90° with the humerus externally rotated (palm up) was normalized. Available online: https://www.dropbox.com/s/mvuzf99ddure2ja/immediate%20post%20injection%2C%20no%20pain%20.mp4?dl=0 (accessed on1 January 2023).
